# Lessons learned from the history of postgraduate medical training in Japan: from disease-centred care to patient-centred care in an aging society

**DOI:** 10.1186/s12960-022-00752-x

**Published:** 2022-06-18

**Authors:** Mari Honda, Nobuaki Inoue, Marco Liverani, Mari Nagai

**Affiliations:** 1 Bureau of International Health Cooperation, National Center for Global Health and Medicine, 1-21-1, Toyama, Shinjuku-ku, Tokyo, 162-8655 Japan; 2 Department of Global Health and Development, Faculty of Public Health and Policy, London School of Hygiene and Tropical Medicine, London, United Kingdom; 3 School of Tropical Medicine and Global Health, Nagasaki University, Nagasaki, Japan

**Keywords:** Universal health coverage, Postgraduate medical training, Patient-centred care, Low- and middle-income countries (LMICs)

## Abstract

**Background:**

Health workers, the core of health service delivery and a key driver of progress towards universal health coverage, must be available in sufficient numbers and distributed fairly to serve the entire population. In addition, the planning and management of the health workforce must be responsive to the changing needs of society, including changes in age structure and epidemiology. Considering these issues, this paper examines in historical perspective the evolution of postgraduate medical training and practice in Japan, from the late nineteenth century to the present.

**Main text:**

When the first medical schools were established in the country towards the end of the nineteenth century, Japan was a largely agrarian society, with a population of about 30 million and an average life expectancy of 30–40 years. During the twentieth century, life expectancy and the national population continued to increase in a context of rapid economic growth. Since the 1980s, another demographic transition has occurred: low fertility rates and an aging society. As a result, the inputs and skills required from health professionals have changed considerably over time, posing new challenges to the national health sector and the management of human resources for health.

**Conclusions:**

The case of Japan offers valuable lessons for other countries experiencing a rapid epidemiological and demographic transition. To provide medical care that meets health priorities in the communities, we must consider not only the training of specialists, but also ensure the availability of a large cadre of physicians who possess basic skills and can provide patient-centred care. Furthermore, the Japanese experience shows that a highly hierarchical system and organisational culture are ill-suited to respond quickly to the changing demands of society.

## Background

Health workers form the core of healthcare service delivery and are a key driver of progress towards universal health coverage (UHC) [1–3]. As such, they must be available in sufficient numbers and distributed fairly to serve the entire population. Furthermore, the planning and management of the health workforce must be responsive to the changing needs of society, including changes in age structure and epidemiology. In the past, most countries experienced a high burden of infectious diseases and a relatively low life expectancy. Today, infectious diseases remain an important concern, but are no longer the leading cause of death in high-income countries and, increasingly, low- and middle-income countries [4]. Owing to advances in medicine and public health, including sanitation and living standards, life-expectancies have increased, allowing for greater chances of developing noncommunicable diseases, such as cardiovascular diseases and diabetes [5]. Along with this epidemiologic transition, improved socioeconomic status, education, and greater access to information sources have raised people’s expectations regarding health services and their right to health. Therefore, a holistic, patient-centred approach, serving the changing needs and expectations of individuals and their communities, has become more important [6]. In turn, this requires adaptive training systems, designed to keep up with epidemiological and social changes [7].

The evolution of postgraduate medical training systems in Japan over the past 150 years provides a critical case to examine these changes and associated challenges. The historical trajectory of Japan illustrates an ‘accelerated model’ of epidemiological transition, where demographic change and the shift from communicable to non-communicable diseases started later than in Western countries, but at a much faster pace [8]. Around the time of establishment of the first medical school in 1857 [9] and towards the end of the nineteenth century, Japan was largely an agrarian society, with a population of about 30 million and an average life expectancy of 30–40 years [10]. During the twentieth century, however, Japan became home to the people with the longest life expectancy, and its population continued to increase in a context of rapid economic growth, surpassing 120 million in 1983 [11]. Since the 1980s, another demographic transition has occurred, characterised by low fertility rates and an increasingly aging society. It was estimated that one in three people in Japan will be 65 or older in 2025, posing new challenges for the national health sector and the management of the health workforce [12].

Several papers have been published in English on health workforce development in Japan, focused on different issues, such as universal health insurance coverage [13] and the shortage of health workforce in rural areas [14, 15]. However, no English-language reviews on the relationship between demographic changes and postgraduate medical training are available, especially from the perspective of the Ministry of Health. Considering this gap in the literature, our purpose herewith is to review the development of medical training in Japan in historical perspective. In the first part of the paper, we examine key developments across three distinctive periods of Japan’s history, from the Meiji period in the late nineteenth century to the present. We then discuss achievements, lessons learned, and remaining challenges, with a view to informing the policy debate in Japan as in other countries, particularly those experiencing a rapid social and demographic transition.

Research for this paper involved a critical literature review of published studies and reports, retrieved in the following online databases: PubMed, Web of Science, Google/Google Scholar, and Ichushi-Web, a bibliographic database of peer-reviewed medical articles in Japanese. To identify relevant contributions, the search terms “medical education”, “medical training”, “school of medicine”, “history” were used in combination with “Japan” and “Japanese”. The searches were conducted either in English or Japanese, depending on the database. In addition to published papers, policy documents and reports were retrieved from the online archives of public organizations, such as the Ministry of Health, Labor and Welfare (MHLW) and the Japan Medical Association. Throughout the selection process, we adopted an wide-ranging approach, including contributions whose main focus was secondary to the central topic of our review, but which presented nonetheless relevant material. The collected documents were examined using a thematic narrative analysis, whereby information on key events and developments in each period was extracted, collated, and triangulated to verify and gain a deeper understanding of emerging findings [16].

## From the Meiji period to World War II

For many centuries, medical practice in Japan was primarily based on concepts and theories derived from China. Western medicine became mainstream only after the Meiji restoration in 1868, the political revolution that ended two centuries of cultural isolation and brought about radical changes to the social and economic life of the country. During the Meiji period (1868–1912), the new rulers recognised the importance of modernisation and opening the country to Western science and technology as part of their plans to build ‘a wealthy country with a strong army’. Hence, physicians from Germany were invited to teach in the University of Tokyo’s medical school, established in 1877 [9]. Graduate students were sent to study in Europe and appointed as lecturers in Japan upon their return. By the century’s end, all professors of medicine at the University of Tokyo were Japanese, and more medical schools were founded in other cities [17].

Medical training in these schools was characterised by a highly hierarchical approach, called *ikyoku*, which remained a defining feature of the medical establishment in Japan throughout the twentieth century. Under this system, based on the German model, the heads of medical school’s departments were responsible not only for the research and teaching programs in their units but also for the placement of graduates to other hospitals for residency [18]. The destination of graduates for residency was decided by the heads of departments, based on their personal relations with hospital directors as well as political and financial considerations [19].

These developments occurred against the backdrop of rapid demographic changes. In 1874, the Japanese population stood at 35 million but had more than doubled in just over 70 years, reaching 72 million in 1945. Simultaneously, the proportion of people living in urban areas increased from 10% in 1898 to approximately 40% by 1940. In this context, new challenges regarding the management of the health workforce emerged. In the 1920s, physicians were concentrated in large cities which had the best medical schools, and where physicians with a private clinic could find ‘good customers’ [20]. As a result, physicians were available only in 30% of all Japanese municipalities. To increase the supply of physicians in rural areas, medical colleges with less stringent admission criteria and shorter programs were established during the 1920s. Between 1938 and 1945, additional medical colleges were established to meet the demand for military doctors, and to replenish physicians who had been drafted in the army [21]. During this period, the priority of the government was to increase the number of physicians; by contrast, the quality of postgraduate medical training received little attention.

## Population growth and epidemiological transition during the economic boom

From the end of World War II (WWII) in 1945 until 1952, Japan was occupied by the Allied Powers. This period was characterized by the development of strong democratic institutions and the introduction of social reforms that laid the foundations for a more egalitarian society. In the health sector, the Public Health and Welfare Bureau (PHW), established by the Supreme Commander for the Allied Powers, rebuilt various aspects of the public health infrastructure and initiated several reforms to improve medical education and clinical practice [17]. Medical colleges that met the new educational standards were upgraded to universities, while others were abolished [19]. Initially, the director of the PHW, Dr. Crawford F. Sams, pushed for the introduction of a 4-year liberal-arts pre-medical education, followed by 4 years of medical–technical training. However, this proposal was rejected by Japanese stakeholders due to the lack of sufficient resources to sustain the program in impoverished post-war Japan. Furthermore, concerns were raised that an 8-year program would not only be prohibitively expensive to the government but it would also limit the number of doctors trained, resulting in a small elite of medical professionals serving only wealthy citizens [22]. Thus, the 6-year medical education program, comprised of 2 years of liberal-arts pre-medical study and 4 years of medical–technical study within the individual medical schools, became the norm in Japan.

Combined with other key reforms of the health sector, the new training system contributed to substantial improvements in health service delivery. In 1961, Japan achieved UHC by mandating all residents to enrol in the social health insurance program [23]. At the same time, the country entered a new phase of demographic transition, becoming the second largest economy in the world in 1968, and further expanding until the 1990s. During this period, the population continued to increase, surpassing 120 million in 1983. As a result, the supply of physicians became inadequate to cover the entire population. In addition, the process of urbanisation intensified as large cities became the centres of industrial capitalism [24], attracting physicians at the expense of rural communities [25].

To address the shortage and maldistribution of physicians, the Ministry of Education and the Ministry of Health and Welfare (MOHW), currently MHLW, implemented three complementary strategies. First, the enrolment capacity of pre-existing medical schools was raised. Second, new medical schools were built nationwide to have ‘one medical school for each prefecture’—a policy goal that was achieved in 1979 [19]. Third, a new private university, the Jichi Medical School, was established in 1972 through an alliance of all 47 prefectures to reduce the shortage of physicians in rural areas [26]. Every year, local governments sponsored two students from their prefectures to attend this school. Successful applicants were exempted from paying the tuition fees as long as they returned to work in their home prefectures after graduation [27].

**Fig. 1:**
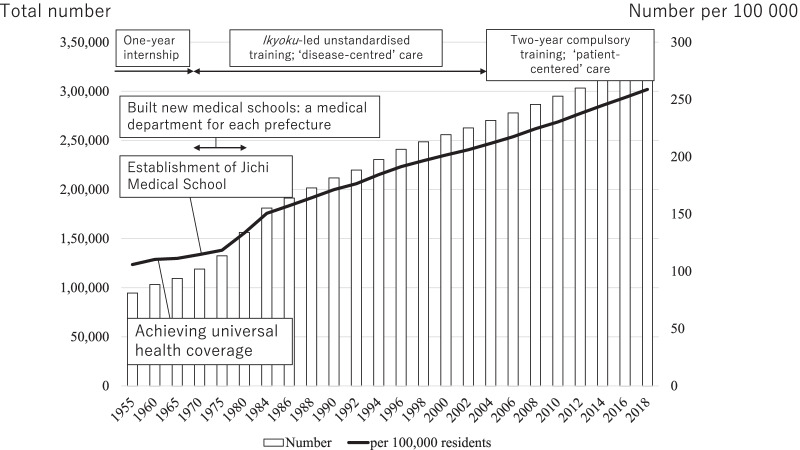
Trends in the total number of physicians and physicians per 100 000 residents in Japan. Original data was retrieved from Ministry of Health, Labour and Welfare, Office of Health Statistics, Policy Management Division. Total Fertility Rate: https://data.worldbank.org/indicator/SP.DYN.TFRT.IN?end=2019&locations=JP&start=1960&view=chart. Population aged 65 and above: https://data.worldbank.org/indicator/SP.POP.65UP.TO.ZS?end=2020&locations=JP&start=1960&view=chart

To a large extent, these interventions were successful. By the mid-1980s, the number of newly certified physicians per year increased from approximately 4000 to 8000 [28]. Consequently, the ratio of physicians per 100 000 population increased from 114 to 164 between 1970 and the late 1980s (Fig. 1) [29]. During the first two decades since its foundation, 792 doctors graduated from Jichi Medical School and returned to work in their home prefectures [27]. In addition, under the *ikyoku* system, many physicians were mandated to work in remote areas [18]. However, while municipalities with a population of over 30 000 gained proportionally more physicians, smaller communities with fewer than 10 000 residents remained underserved [25]. Thus, the maldistribution of physicians was not fully addressed.

Furthermore, new challenges arose from the rapid epidemiological transition. Immediately after the end of WWII, malnutrition and communicable diseases were the most important public health concerns in Japan. After the 1950s, however, non-communicable diseases became the primary cause of death and disability. Between 1960 and 1980, cancer and cardiovascular diseases increased by 72% and 81%, respectively, requiring new interventions and national efforts to address this problem [30]. Notably, the National Cancer Centre was established in the early 1960s, and the National Cardiovascular Centre followed in 1977 to train specialised physicians, provide advanced care, and pursue cutting-edge research [31].

During this period, the MOHW also attempted to improve the general training program in medicine and redress the maldistribution of physicians. A mandatory 1-year internship had been introduced in 1948 under the American occupation; however, the inadequacy of the training curriculum and the lack of financial security during the internship period caused great dissatisfaction among graduates and students, and the system was abolished in 1969. By contrast, the *ikyoku* system was welcomed by graduates, because it enabled them to develop highly specialised clinical and research skills through postgraduate training. Each *ikyoku* covered specific diseases and clinical research.

## Struggling to adjust postgraduate medical training systems to an aging society

**Fig. 2:**
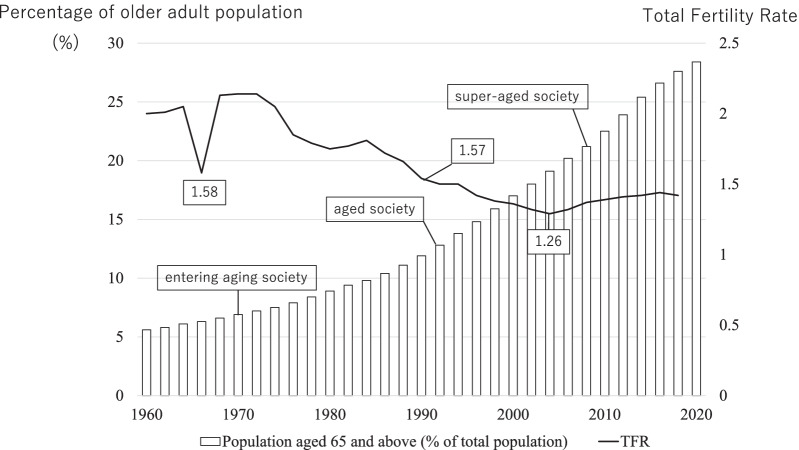
Trends in the percentage of the older adult population and total fertility rate in Japan. Original data was retrieved from World Bank Open Data

Since the 1970s, Japan experienced another demographic change characterised by an increasingly aging society. In 2007, Japan became a ‘super-aged’ society with more than 21% of the population over 65 years (Fig. 2). Recently, it was estimated that older adults will comprise approximately 30% of the population by 2025 [32]. A combination of different factors has led to this unusually rapid demographic change, including improvements in the living environment, healthier diet and nutrition, and advances in medical technology leading to a decrease in infant deaths and a corresponding increase in life expectancy [33]. Simultaneously, a decline in marriage rates, increasing economic and childcare burden, and infertility have resulted in a sharp decline in birth rates [34]. In 1990, the MOHW announced that the total fertility rate (TFR) had reached 1.57, the lowest rate in recent Japanese history (Fig. 2). Until then, such a low rate was recorded only in 1966 as many Japanese families chose not to have children due to a superstition known as *Hinoe-Uma* (‘fire horse’), which predicted that women born in that particular year would have a bad personality [35]. In the new millennium, the TFR fell further, reaching 1.26 in 2005, and has remained low ever since [34].

As a result of these changes, the need to train physicians with comprehensive clinical knowledge and skills to provide care for older adults, who often have multiple health-related problems, became more urgent. However, after the abolition of the mandatory 1-year internship program in 1969, a standardised postgraduate medical training immediately after graduation from medical school was not established [36]. Each hospital conducted its own training with unique curriculum and its quality varied [37]. Since the *ikyoku*-led postgraduate medical training mainly aimed at acquiring specialised knowledge and skills, the various needs of older adults, such as prevention of lifestyle-related diseases, were left unaddressed [37].

In this context, in 1978 the MOHW issued a notice titled ‘Implementation of postgraduate medical training with primary care’, which allowed graduates to choose either ‘rotational training’ at several departments closely related to their future specialties, or the long-standing ‘*ikyoku* training for specialties’ in one department [38]. Furthermore, a ‘general practice course’ was added in 1985, which involved rotation through four different specialties: internal medicine, surgery, paediatrics, and emergency medicine. The MOHW attempted to increase the incentives for hospitals to offer this course [39]. However, these policy interventions could not change the firmly established *ikyoku* postgraduate training system. According to a survey of 42 national medical school-affiliated hospitals designated for training in 1987, only 16 hospitals had a ‘rotational training’ curriculum and only 5 hospitals had a ‘general practice course’ [40].

Towards the end of the 1990s, a more radical change of the *ikyoku* system was triggered by two shocking cases of medical malpractice. First, a patient with heart disease and another with lung cancer were mistaken and given the wrong surgeries at a university hospital. In the other case, a postoperative patient was mistakenly injected with disinfectant, instead of anticoagulant, resulting in death [41]. This led to increased awareness in Japanese society of patients’ rights, patient-centred care, and medical safety [42]. As a result, the number of reports and lawsuits concerning medical malpractice increased steeply since the early 2000s [42]. In addition, concerns emerged that the *ikyoku* system was partly responsible for these cases of medical malpractices because of the lack of transparency due to its patriarchal system [43].

Amidst these concerns, there was increasing pressure to transition from a disease-centred to a patient-centred model of care. In 1997, the Ministry of Education, Culture, Sports, Science and Technology and the MHLW collaborated with the Postgraduate Medical Training Council to introduce standardised training at medical schools, university hospitals, and hospitals [44]. As a result, in 2004 the 2-year compulsory postgraduate training was implemented under the amended Medical Practitioners Act and the Medical Service Act. Postgraduate training became mandatory at designated training hospitals, and was undertaken under the guidance of MHLW-certified medical instructors [45]. Graduates were expected to acquire an adequate understanding of primary care and embrace a holistic approach by treating the patient as a ‘whole’ person instead of focusing purely on an illness or diagnosis [45]. In addition, hospitals designated for postgraduate training, including university hospitals, were required to pay sufficient wages to allow graduates to focus on their training. Since the introduction of this approach, the *ikyoku* system slowly weakened its control over postgraduate training for specialties [46]. Recently, some medical schools have also introduced interprofessional education (IPE) to prepare medical students for multidisciplinary teamwork in their future clinical work. These curricula are not standardized at the national level yet, although the Model Core Curriculum for medical schools recognises that the ability to engage in multidisciplinary collaboration in health and social care is a basic skill of medical personnel [47].

Despite much progress, critical issues in the Japanese training system remain. Importantly, there is a longstanding gender bias as fewer women than men are routinely admitted to medical schools. This bias has somehow been justified with the assumption that female physicians cannot allocate as much time as men to their profession as they need to fulfill their responsibilities in the household [48]. However, this assumption reflects deep-seated issues around gender inequality in Japanese society, which ranks 120 out of 156 in the World Economic Forum’s 2021 Gender Gap Index [49]. In recognition of this, some changes to the governance of medical practice are being made. For example, the recent physicians’ labour reforms of MHLW, based on the 2017 National Action Plan for the Realization of Work Style Reform, focuses on limiting overtime [50]. It is expected this reform will also foster a more women-friendly work environment and break gender stereotypes [51], although it is still too early to assess the policy outcomes.

In sum, the past two decades have been characterised by the MHLW’s efforts to reform the postgraduate medical training system and strengthen the government’s capacity to enhance the quality and relevance of medical practice. These efforts are characterised by a shift in the medical training system, from an exclusive focus on advanced and specialised medical care under the leadership of *ikyoku*, to an emphasis on the importance of comprehensive clinical knowledge and multidisciplinary teamwork skills. As we have seen, this shift has been triggered by the need to address an increasingly aging society and, at the same time, deliver a health service focused on the needs, rights, and expectations of the patients.

## Discussion

The history of the Japanese postgraduate medical training highlights the importance of achieving a balance between specialisation in one field of medicine and the ability to provide comprehensive and holistic care. From the Japanese experience, several lessons can be drawn for other countries undergoing rapid epidemiological transition. First, the large-scale production of specialised physicians is not enough to respond to the needs of an aging society, where patients are often affected by complex chronic health problems. Indeed, a recent study of health-seeking behaviour in Japan found that almost 80% people had some symptoms, but only 1.3% of them required specialised medical care [52]. This finding indicates that only a minority of patients in an aging society require advanced medical care, while the majority can be treated by generalists at a primary care facility. Care provided by a generalist has the potential to reduce costs to the health sector while maintaining quality [53] and improving the health and life expectancy of citizens [54, 55].

The second lesson is about challenges to policy reform in a highly hierarchical medical establishment. The *ikyoku* system, focused on the formation of specialised physicians, was characterised by a rigid top-down organisational culture and governance structure. This approach, which held significant sway for more than 100 years, has made it difficult to introduce adaptive policy reforms to respond to the changing public health priorities and needs of society. Recognising the limitation of this model, the Japanese government has recently introduced various initiatives to reform medical education—shifting to a more holistic, community-oriented educational environment. Furthermore, the MHLW has initiated decentralization policy to meet the health needs of rural communities, establishing the Regional Medical Support Centres in each prefecture [56]. However, this has been a long and slow process due to the persistence of the *ikyoku* system and the initial reluctance of the academic establishment to initiate policy reform.

Third, the unacceptable gender bias still exists as one of the greatest weaknesses of the Japanese medical system. The hierarchical system has disadvantaged female physicians for years in spite of the fact that gender diversity in the health workforce may increase the utilization of health services and patient satisfaction [57]. Regrettably, the gender gap in the medical education system is reflected in clinical and academic medicine [58]. Continuous efforts are required to address these gender gaps and their negative effects on patient-centred care.

Fourth, the debate about the feasibility of an 8-year medical education program during the post-war occupation period shows the critical importance of country ownership and sustainable policy development in an impoverished country. This is a critical lesson for LMICs and donors alike. When LMICs face strong recommendations from international partners pushing for investment in a high-level medical education system, consideration should be given to what the country can afford and how greater equity and distribution of limited resources can be ensured in the longer term. In fact, the establishment of an ‘elite’ education system could result in the migration of local physicians to wealthier countries, since their education is not aligned with the economic condition in their own country [59].

Fifth, regardless of the national context or economic status, there is a general tendency for medical students and physicians to pursue higher and narrower specialised knowledge and skills, since these are intellectually more stimulating and offer better career prospects [60]. By contrast, the low prestige and academic profile of primary care are strong disincentives for becoming a general practitioner [61]. One possible strategy to redress this specialists-oriented culture and train physicians with comprehensive clinical knowledge is the integration of IPE in undergraduate education [62]. Furthermore, some countries have established postgraduate training curricula that include comprehensive medical care along with advanced specialisation. In the United Kingdom and Australia, for example, a basic foundational training program is compulsory before proceeding to specialty training [63]. This approach has also been adopted in Japan, where the training program includes internal medicine along with general surgery and paediatrics.

The importance of training medical specialists is obvious and well recognized globally. However, the focus on medical specialization must not result in a reduced supply of general practitioners, who can provide essential health services in the communities [64]. In Africa, for example, a number of countries have introduced or strengthened specialty training as a strategy to reduce the migration of medical school graduates to other countries with more advanced training systems [65, 66]. While this strategy has been proved to increase retention, the case of post-war Japan documents that an exclusive emphasis on specialization has the potential to produce unintended and undesirable consequences, such as diverting scarce human resources from primary care and health priorities in the communities. Ultimately, these should remain the foundations of any reforms of the medical education system, in Japan and in other countries [67, 68].

### Study limitations

This paper relied only on a review of the literature and did not include other information sources. In future studies on this topic, interviews with stakeholders would be particularly useful to triangulate findings and elicit information on the policy process that is not available in the public domain. In addition, further research on the history of medical education in Japan could consider other categories of health professionals, such as nurses and midwives.

## Conclusions

In each country, the medical training systems must be able to adapt to the changing population needs during demographic and epidemiological transitions. At any points in time, however, a large cadre of physicians who possess basic skills and can provide patient-centred, primary care is needed. We hope that 100 years of experiences in Japan can help other countries as they adjust their health workforce development plans to meet present and future health challenges.

## Data Availability

Data sharing is not applicable to this article as no data sets were generated or analysed during the current study.

## Abbreviations

IPE: Interprofessional education
LMICs: Low- and middle-income countries
MHLW: Ministry of Health, Labour and Welfare
MOHW: Ministry of Health and Welfare
PHW: Public Health and Welfare Bureau
TFR: Total fertility rate
UHC: Universal Health Coverage
WWII: World War II
